# Plasma lipidome variation during the second half of the human lifespan is associated with age and sex but minimally with BMI

**DOI:** 10.1371/journal.pone.0214141

**Published:** 2019-03-20

**Authors:** Matthew Wai Kin Wong, Nady Braidy, Russell Pickford, Fatemeh Vafaee, John Crawford, Julia Muenchhoff, Peter Schofield, John Attia, Henry Brodaty, Perminder Sachdev, Anne Poljak

**Affiliations:** 1 Centre for Healthy Brain Ageing, School of Psychiatry, Faculty of Medicine, University of New South Wales, Sydney, Australia; 2 Bioanalytical Mass Spectrometry Facility, University of New South Wales, Sydney, Australia; 3 Systems Biology, School of Biotechnology and Biomolecular Sciences, Faculty of Science, University of New South Wales, Sydney, Australia; 4 School of Medicine and Public Health, University of Newcastle, Newcastle, Australia; 5 Neuropsychiatric Institute, Euroa Centre, Prince of Wales Hospital, Sydney, Australia; 6 School of Medical Sciences, Faculty of Medicine, University of New South Wales, Sydney, Australia; Nathan S Kline Institute, UNITED STATES

## Abstract

Recent advances in mass spectrometry-based techniques have inspired research into lipidomics, a subfield of ‘–omics’, which aims to identify and quantify large numbers of lipids in biological extracts. Although lipidomics is becoming increasingly popular as a screening tool for understanding disease mechanisms, it is largely unknown how the lipidome naturally varies by age and sex in healthy individuals. We aimed to identify cross-sectional associations of the human lipidome with ‘physiological’ ageing, using plasma from 100 subjects with an apolipoprotein E (APOE) E3/E3 genotype, and aged between 56 to 100 years. Untargeted analysis was performed by liquid chromatography coupled-mass spectrometry (LC-MS/MS) and data processing using LipidSearch software. Regression analyses confirmed a strong negative association of age with the levels of various lipid, which was stronger in males than females. Sex-related differences include higher LDL-C, HDL-C, total cholesterol, particular sphingomyelins (SM), and docosahexaenoic acid (DHA)-containing phospholipid levels in females. Surprisingly, we found a minimal relationship between lipid levels and body mass index (BMI). In conclusion, our results suggest substantial age and sex-related variation in the plasma lipidome of healthy individuals during the second half of the human lifespan. In particular, globally low levels of blood lipids in the ‘oldest old’ subjects over 95 years could signify a unique lipidome associated with extreme longevity.

## Introduction

In recent times, mass spectrometry-based lipidomics techniques have emerged as important technological platforms enabling the identification of hundreds to thousands of lipids in plasma and tissue extracts [1]. Lipids play an important role in cellular metabolism by maintaining structural integrity, and acting as signalling molecules, and may have important implications in health and disease. Lipidomics is commonly used to identify and quantify metabolic changes associated with age related disorders such as cardiovascular disease, diabetes, metabolic syndrome [2], cancer [3], and neurological disorders, such as Alzheimer’s disease [4–6]. It therefore forms a strong and relatively novel starting point for the study of disease mechanisms or disease-related biomarkers. However, before investigating pathological changes to the lipidome involved in disease states, it is important to understand how the human lipidome naturally varies in normal healthy individuals based on anthropometric variables such as age, sex and BMI. This knowledge is not only of intrinsic interest to better understand the biology of ageing, but will also aid in appropriate experimental design and subject selection to address major research questions.

In this study, we focused on blood as an important source of biomarkers and which is already widely used for both clinical and research purposes [6, 7]. Furthermore, venepuncture is a relatively safe, minimally invasive procedure and blood is easy to collect (even for repeat analyses) and store and therefore represents a convenient medium for investigating lipidomics [6, 8].

We applied a recently described technique [9] to extract lipids from human plasma samples. Plasma lipid extracts were then analysed using liquid chromatography electrospray ionisation tandem mass spectrometry (LC-ESI MS/MS) to investigate how major lipid classes were altered as a function of age. We also wished to avoid the minor *APOE* allele variants ε2 and ε4, which are frequently reported disease modulators [10, 11], and given the known *APOE* association with plasma lipids, we particularly wished to eliminate this confounder. We therefore selected exclusively *APOE* ε*3* homozygous individuals to explore relationships between plasma lipids and other anthropomorphic variables such as sex, BMI and cholesterol levels (HDL-C and LDL-C). In younger age groups (aged 20 to 50 years), it has previously been reported that plasma lipid levels are associated with age [8, 12], sex [8, 13] and BMI [14, 15]. We therefore hypothesised that lipid profiles in normal healthy individuals will be associated with age, sex and BMI during the second half of the human lifespan, and these relationships will be different in the ‘oldest old’ who exemplify exceptional ageing.

## Materials and methods

### Subject cohorts

Our study sample comprised cognitively ‘healthy’ subjects (i.e. without dementia or MCI) aged between 56–100 years (*n* = 100) enrolled in three independent population ageing studies, which included the Sydney Memory and Ageing Study (MAS, *n* = 40), the Sydney Centenarian Study (SCS, *n* = 20) and the Hunter Community Study (HCS, *n* = 40). These longitudinal studies involve collection of patient data, including results from blood chemistry, MRI, neuropsychiatric assessment/cognitive tests, and medical exams performed over several waves, at an interval of 2–3 years [16–18]. Since no single study can provide plasma samples from subjects over the entire age range required, plasma was taken from wave 1 of the three independent cohorts in order to capture the age range of interest and enable comparison between the youngest and oldest ages. In particular, the HCS sample comprises subjects aged from 56 to 75, residing in Newcastle, New South Wales, while the MAS sample comprises subjects aged from 75 to 90 years of age, and the SCS sample comprises exceptionally long-lived subjects aged 95 through 100 years. The study protocol for each cohort has been previously published [16–18].

The inclusion criteria included subjects enrolled in MAS, SCS or HCS, and with *APOE E3/E3* genotype. Participants who were of non-English speaking background, or who had mini mental state examination (MMSE) scores<20, or had significant neuropsychiatric disorders, cancer, or had cardiovascular complications were excluded from this study.

### Experimental design

To ensure adequate distributions of age, sex and BMI in the sample, as well as in joint distributions of these variables, our sample was selected as follows. From the three studies, 20 participants were selected in each age decade (55–64, 65–74, 75–84, 85–94, >94), and with approximately equal numbers of males and females in each age group. Subjects were also selected so that approximately half of the subjects within each age group, and also within each sex, were in “high” and “low” BMI ranges. (These ranges were chosen so that subjects in the low-BMI group were those with BMI in the so-called “normal” range 18–25, and those in the high-BMI group (BMI >28) corresponding approximately to those classified as obese (defined as BMI>29).)

### Ethics approval

The SCS and MAS were approved by the Ethics Committees of the University of New South Wales and the South Eastern Sydney and Illawarra Area Health Service (ethics approval HC12313 and HC14327, respectively). The HCS was approved by the University of Newcastle and Hunter New England Human Research Ethics Committees (HREC 03/12/10/3.26). All work involving human subjects was done in accordance with the principles of the Declaration of Helsinki of the World Medical Association. Informed consent was obtained from all participants/and or guardians.

### Lipid-lowering medication

To lessen the potential effects of lipid-lowering medication on overall plasma lipids [19], we aimed to minimise the number of the subjects on lipid-lowering medication. Nevertheless, based on the pool of subjects available some subjects were included who were on lipid-lowering medication at the time of blood extraction. In all, 26 out of 100 subjects (14 males, 12 females) were on lipid-lowering medication, and half of these subjects were aged 85 years or older.

### Plasma collection, handling and storage

Blood collection, processing and storage were performed under strict conditions to minimize pre-analytical variability [6, 20]. Fasting EDTA plasma was separated from whole blood within 2–4 hours of venepuncture and immediately stored at -80°C prior to bio-banking. Samples then undergo a single freeze thaw cycle for the purpose of creating aliquots, which minimizes subsequent freeze thaw cycles for specific experiments. EDTA plasma was chosen as anticoagulant since it chelates divalent metals, thereby protecting plasma constituents from oxidation, which is particularly important for lipids. Thereafter, lipid extractions were performed within 15 minutes of freeze thawing and extracts stored at -80°C.

### Targeted assays of plasma lipids

Plasma total cholesterol, LDL-C, HDL-C and TG were measured by enzymatic assay at SEALS pathology (Prince of Wales Hospital) as previously described [21], using a Beckman LX20 Analyzer with a timed-endpoint method (Fullerton, CA). LDL-C was estimated using the Friedewald equation (LDL-C = total cholesterol—HDL-C—triglycerides/2.2).

### Internal standards

Internal standards were purchased from *Avanti* (Alabaster, United States). An equal volume (100pmol/10μL) of internal standards for each lipid class was added to samples and controls prior to lipid extraction. Internal standard peak areas were used to normalise plasma lipid peak areas and to correct for matrix effects and run to run variability [22]. The deuterated internal standards used were: Cer(d18:1/12:0), SM (d18:1/12:0), *d*_*5*_TG(d16:0/18:0/16:0) and *d*_*5*_DG(19:0/19:0). The odd-carbon chain structural analog internal standards were cholesterol ester (CE) CE(19:0), phosphatidylcholine PC(19:0/19:0), phosphatidylethanolamine PE(17:0/17:0), and lysophosphatidylcholine (LPC) LPC(19:0).

### Lipid extraction from plasma: Single phase 1-butanol/methanol

We added 10 μL of internal lipid standards mixture (described above) to 10 μL plasma in Eppendorf 0.5 mL tubes. 100μl of 1-butanol/methanol (1:1 v/v) containing 5 mM ammonium formate was then added to the sample. Afterwards, samples were vortexed for 10 seconds, then sonicated for one hour. Tubes were centrifuged at 13,000 g for 10 minutes. The supernatant was then removed via a 200 μl gel-tipped pipette into a fresh Eppendorf tube. A further 100μl of 1-butanol/methanol (1:1 v/v) was added to the white pellet to re- extract any remaining lipids. The combined supernatant was dried by vacuum centrifugation and resuspended in 100 μl of 1-butanol/methanol (1:1 v/v) containing 5 mM ammonium formate and transferred into 300 μl Chromacol autosampler vials containing a glass insert. Samples were stored at -80° C prior to LC-MS analysis.

### Liquid chromatography/ mass spectrometry

Lipid analysis was performed by LC ESI-MS/MS using a Thermo QExactive Plus Orbitrap mass spectrometer (Bremen, Germany). A Waters ACQUITY UPLC CSHTM C18 1.7um, 2.1x100mm column was used for liquid chromatography at a flow rate of 260 μL/min, using the following gradient condition: 32% solvent B to 100% over 25 min, a return to 32% B and finally 32% B equilibration for 5 min prior to the next injection. Solvents A and B consisted of acetonitrile:MilliQ water (6:4 v/v) and isopropanol:acetonitrile (9:1 v/v) respectively, both containing 10 mM ammonium formate and 0.1% formic acid. Product ion scanning in positive and negative ion modes were performed to maximise the breadth of lipid species coverage. Sampling order was randomised prior to analysis.

### Alignment and peak detection/analysis

The raw data was aligned, chromatographic peaks selected, specific lipids identified and their peak areas integrated using Lipidsearch software v4.1 (Thermo Fischer Scientific, Waltham MA). Data were then exported to an Excel spreadsheet for manual processing and statistical analysis. The raw abundances (peak areas) were normalised by dividing each peak area by the raw abundance of the corresponding internal standard for that lipid class e.g. all ceramides were normalised using Cer(d18:1/12:0). The intra-assay coefficient of variation (CV) was calculated by dividing the standard deviation of the normalised abundances by the mean across lipid species. Lipid ion identifications were filtered using the LipidSearch parameters rej = 0 and average peak quality>0.85. Furthermore, identifications with CV<0.4 from repeated injections of quality control plasma samples were included (see supporting methods in S1 Appendix).

### Data analysis

Normalised peak areas for each lipid were exported to IBM SPSS 24.0 and R. Sums of normalised abundances for each lipid class/subclass were calculated and compared across age, sex and BMI (see S1 Table for list of lipid classes and subclasses examined). The Shapiro-Wilk test was performed to assess the normality of the distribution of lipid levels. If distributions were found to be non-normal, either non-parametric statistical procedures were used, or else variables log-10 transformed to more closely approximate the normal distribution (in the case of ordinary least squares regression). Since these variables tended to violate normality (p<0.05), the Mann-Whitney U test (non-parametric equivalent of Student’s t-test) was used to compare lipid class abundances between pairs of groups, such as by sex, lipid lowering medication usage, and binary BMI group, while the Kruskall-Wallis test (non-parametric equivalent of ANOVA) was used to examine the statistical significance of relationships with categorical variables with more than two levels, particularly differences in lipid abundance by age decade. Pearson’s product moment coefficient was used to calculate the correlations between normalised abundances of lipid classes and LDL-C, HDL-C and total cholesterol. Hierarchical clustering was used to group lipid classes with similar patterns of correlations with other lipid classes. The lipid classes were then ordered by group in the correlation matrices. The agglomeration method used in hierarchical clustering was “complete linkage” with Euclidean distance metric. This method identifies clusters of lipid classes that have similar correlations with other lipid classes.

Factor analysis was applied in order to reduce multiple dependent variables (lipid classes) into a smaller set of factors that combines the maximum common variance of these variables. Individual lipid classes were factor analysed using principal component analysis and direct oblimin rotation with Kaiser normalisation, yielding two factors, explaining 64.7% of the variance for the entire set of variables. The component scores were used as dependent variables for further regression analyses. Ordinary least squares linear regression was used to examine the effects of age, sex and BMI on normalised lipid abundances. A product term representing the interaction between age and sex was also included in the models. Thus we modelled normalised lipid abundance as a linear combination of age, sex, BMI and age by sex interaction. Age was centred at 75 years (i.e. true age minus 75 years) to reduce collinearity between age and the interaction between age and sex. For these analyses, those dependent variables found to be non-normal were log10-transformed to reduce skewness and so more closely approximate the normal distribution. In these analyses, sex was coded as male = 0 and female as 1. So, with the interactions of age and sex included in the models, the regression coefficient of age represents the relationship for males. We then repeated this regression analysis by recoding the sex as male = 1 and female = 0 in order to isolate the effect of age on lipids in females only, thus providing analyses of associations between lipid abundances and age, stratified by sex. We corrected levels of statistical significance for multiple testing using the Benjamini-Hochberg false discovery method [23].

### Lipid shorthand notation

For each lipid class/subclass analysed, specific lipids are named according to LIPID MAPS convention [24], with slight modification to denote summation of lipids of a particular class/subclass. We have applied the following shorthand notation: Cer(d18:1/X) refers to the sum of all Cer with an 18:1 fatty acid in the sn-1 position, while CE(18:X) refers to the sum of all CE with an 18 carbon chain length.

## Results

### Participant demographics

A summary of the demographics and lipid profile of the five age decades used in this study is shown in Table 1. The overall statistical test for inequality of years of education was not significant (p>0.05). The mini-mental state examination (MMSE) score was lower for the 95+ age group compared to younger age groups (p<0.01, Kruskall Wallis test), except for the 65–75 age group. The waist-hip ratio (WHR) was reported for subjects in the HCS and MAS studies (data unavailable for SCS) and was not significantly different between age groups. The levels of LDL-C, HDL-C, total cholesterol and TGs were also not significantly different between age groups (p>0.05, Kruskall Wallis test), nor cohorts (S2 Table).

**Table 1:** Patient characteristics and lipid profiles by age decade.

	56-<65 yrs	65-<75 yrs	75-<85 yrs	85-<95 yrs	95+ yrs	Chi-square
N	21	19	20	20	20	N/A
Age	58.5 (2.6)	68.4 (2.3)	79.1 (2.9)	87.1 (1.9)	96.6 (1.4)	94.8*
BMI	28.9 (5.2)	27.5 (5.3)	28.3 (5.0)	27.9 (5.2)	26.6 (5.8)	2.459
Lipid-lowering medication	2 (9.5%)	5 (26.3%)	6 (30%)	8 (40%)	5 (25%)	2.029
Years of Education	12.3 (1.9)	11.4 (2.3)	11.25 (3.8)	10.6 (4.3)	10.1 (2.8)	9.076
MMSE score	28.7 (1.1)	27.8 (1.2)	29.1 (0.8)	29.3 (0.73)	26.0 (3.5)	25.94*
WHR	0.89 (0.11)	0.88 (0.08)	0.92 (0.06)	0.94 (0.11)	N/A	3.951
LDL-C (mmol/L)	3.37 (0.79)	3.06 (1.0)	2.85 (0.9)	3.22 (1.19)	2.94 (1.06)	2.840
HDL-C (mmol/L)	1.38 (0.35)	1.37 (0.38)	1.4 (0.29)	1.38 (0.39)	1.47 (0.38)	0.539
Total Cholesterol (mmol/L)	5.39 (0.7)	5.13 (1.0)	4.77 (1.0)	5.21 (1.32)	4.93 (1.10)	4.384
Triglycerides (mmol/L)	1.33 (0.97)	1.29 (0.82)	1.09 (0.49)	1.34 (0.75)	1.13 (0.42)	1.348

Abbreviations: body mass index (BMI), mini-mental examination (MMSE), waist-hip ratio (WHR), low density lipoprotein cholesterol (LDL-C), high density lipoprotein cholesterol (HDL-C).

Values represent mean (SD).

* p<0.05

Kruskall Wallis test was used for all variables except the use of Lipid-lowering medications, in which case the Chi-square test for equality of proportions was used.

Comparing subjects by sex (S3 Table), age, BMI and years of education of males and females in our study were not statistically different (p>0.05, Mann-Whitney U test), though WHR was significantly higher in males (p<0.001). Females also had higher LDL-C, HDL-C and total cholesterol levels compared to males (p<0.05).

### Effect of lipid-lowering medication on lipids

**Fig 1:**
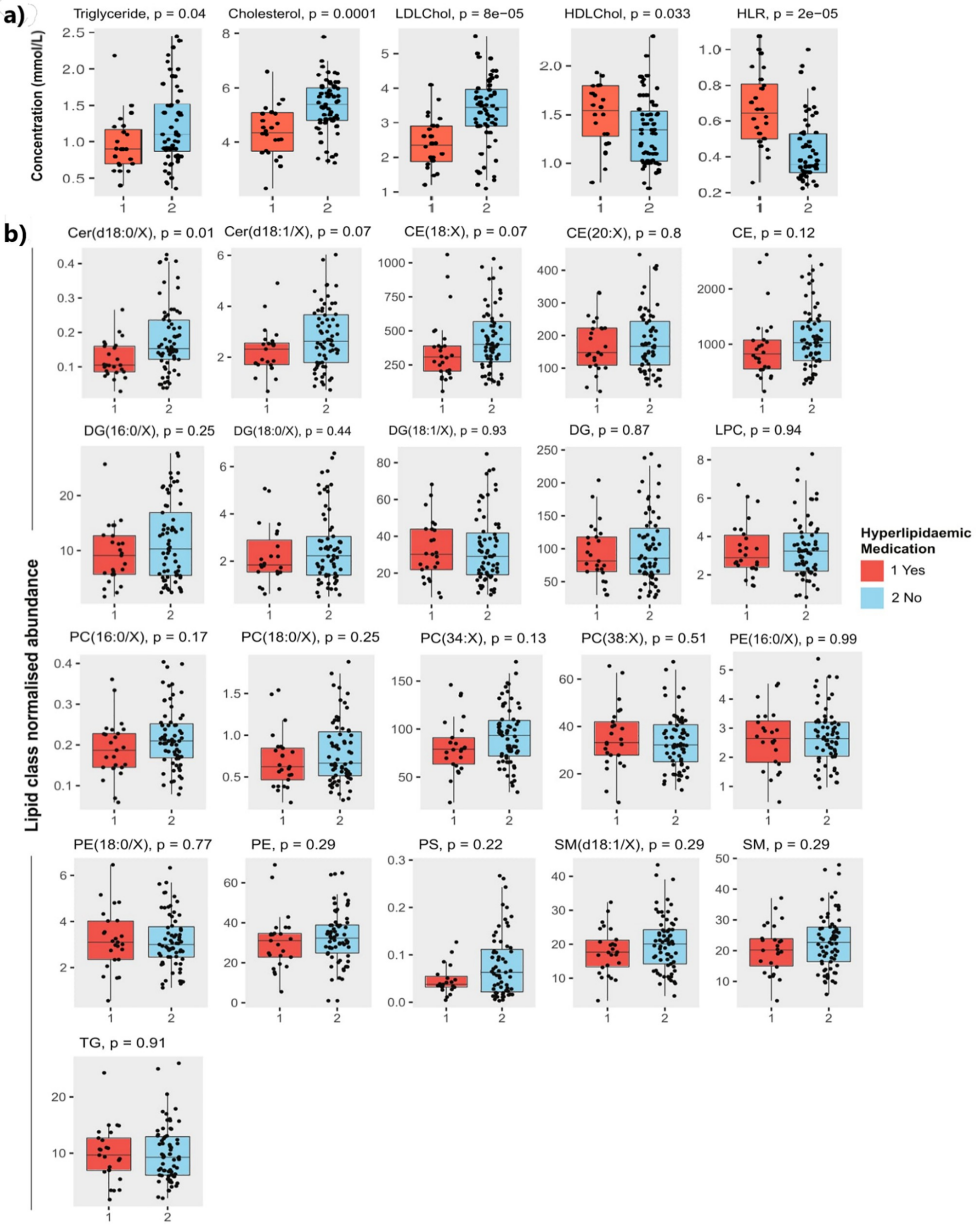
Lipid-lowering medications and plasma lipids. Effect of lipid-lowering medication usage on a) concentrations (mmol) of cholesterol, LDL-C and HDL-C, triglycerides and HLR (*p<0.05, Mann-Whitney U test) and (b) on normalised lipid abundances for lipid classes.

Subjects on lipid-lowering medication had lower total cholesterol, LDL-C, and triglycerides (p<0.05, Mann-Whitney U-test). By contrast, these subjects had higher HDL-C levels, and significantly higher HDL-C to LDL-C ratio (HLR) (Fig 1A), and had lower levels of Cer(d18:0/X) (dihydroceramides) and trended lower Cer and CE(18:X) levels (p = 0.07) compared to their drug naïve counterparts (Fig 1B). There were no other significant differences in the abundance of other lipid classes by lipid-lowering medication.

### Correlations of LDL-C, HDL-C and total cholesterol with lipid classes

**Fig 2:**
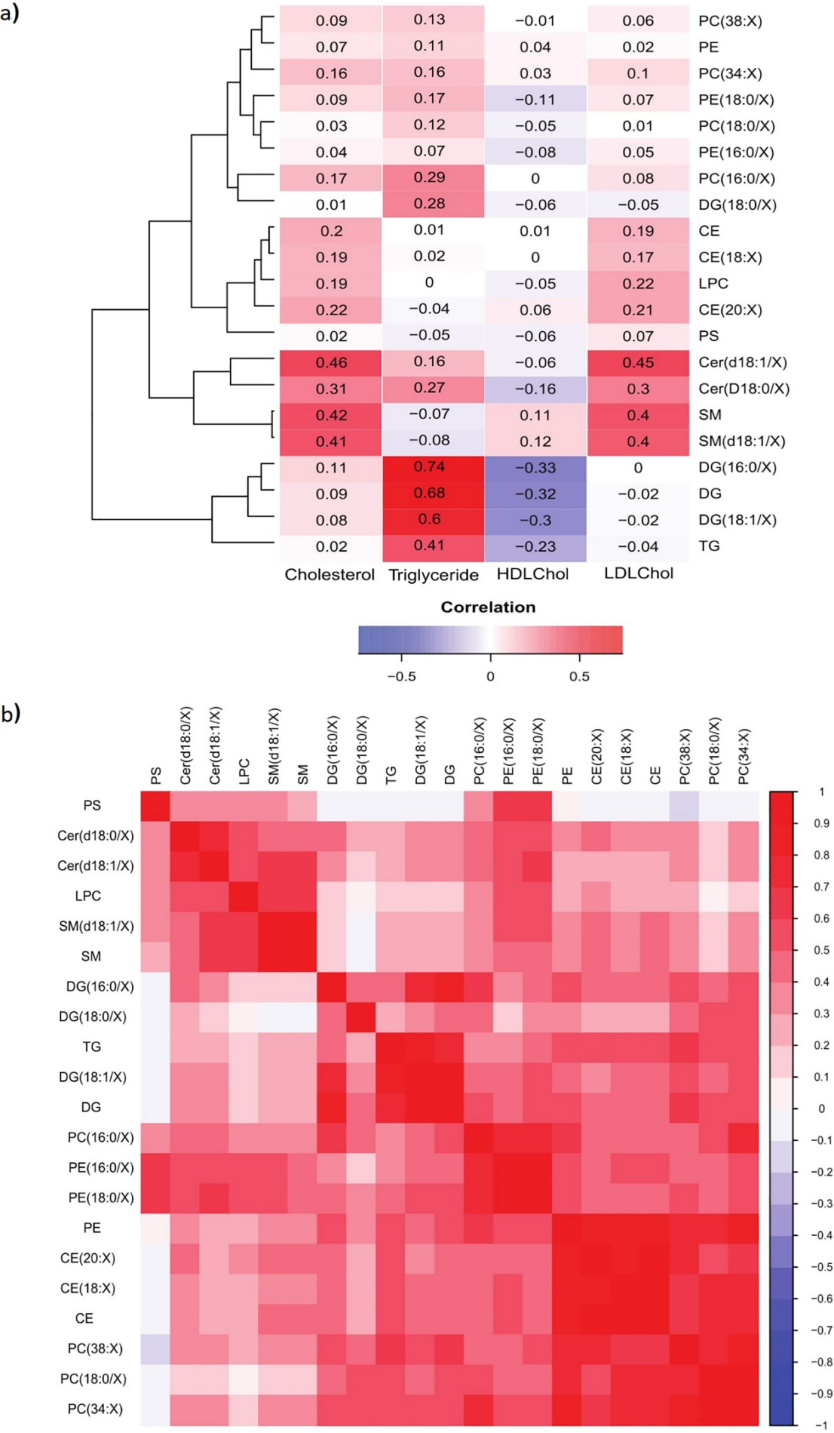
Correlation matrices of traditional lipid measures and lipid classes. (a) Correlation matrix of cholesterol, LDL-C, HDL-C and triglyceride levels with LC-MS measured lipid classes; numbers show 2-digit rounded correlation values; dendrograms represent the hierarchical clustering of lipid classes according to their correlation measures. All correlations above r = 0.30 are considered significant at the p = 0.05 level. (b) Correlation matrix of lipid classes with each other ordered by hierarchical clustering to group together the correlated lipid classes. Heatmap scale represents correlation strength, with red and blue for positive and negative correlations respectively. All correlations above r = 0.30 or below -0.30 are considered significant at the p = 0.05 level.

Pearson correlations of LDL-C, HDL-C and total cholesterol with lipid classes are shown in heatmap form (Fig 2A). LDL-C and total plasma cholesterol were significantly and positively correlated with total Cer(d18:1/X) species, as well as total SM(d18:1/X) species (r = 0.45 and 0.40 for LDL-C with Cer and SM respectively, p<0.001, and r = 0.46 and 0.42 for cholesterol with Cer and SM respectively, p<0.001). All other correlations were not significant (p>0.05). The only significant correlations of HDL-C with lipid class were negative correlations with DG and TG, (r = -0.33 and r = -0.23, respectively, p<0.05). TGs were significantly correlated with total DG levels (r = 0.68, p<0.001). Most lipid subclasses were positively inter-correlated, especially within a class, except for PS, which was not associated with DG, TG or most phospholipids (Fig 2B).

### Principal component analysis of lipid classes

Principal component analysis yield two factors (see Table 2 for the factor pattern matrix). The correlation between the two factors was 0.40. The first factor (FAC1) is likely linked to LDL-C and ApoB particles, since the factors that loaded strongly to it included Cer, SM, as well as LPC and PE, while the second factor (FAC2) is likely linked to TG-rich VLDL due to high loadings by DG, TG and PC. These two factors explained 51.6% and 13.0% of the variance respectively.

**Table 2:** Principal component analysis: Component pattern matrix.

	Component		h^2^
Lipid Class	1	2	
SM(d18:1/X)	**.940**	-.152	0.793
LPC	**.871**	-.208	0.656
Cer(d18:1/X)	**.809**	.051	0.689
Cer(d18:0/X)	**.783**	.075	0.666
PE(16:0/X)	**.698**	.326	0.776
CE(20:X)	**.683**	.204	0.619
PE(18:0/X)	**.672**	.395	0.82
CE(18:X)	**.666**	.239	0.689
PS	**.521**	-.040	0.256
DG(18:0/X)	-.291	**.871**	0.641
DG(16:0/X)	.041	**.821**	0.703
DG(18:1/X)	.151	**.760**	0.692
PC(18:0/X)	.215	**.639**	0.565
TG	.348	**.586**	0.627
PC(16:0/X)	.354	**.537**	0.566

Factors were extracted by principal component analysis and solution rotated by to simple oblique structure with Kaiser normalisation. Loadings are displayed for each factor/component. Percentage of variance explained = 64.7%. h^2^ = communalities (proportion of each variable’s variance explained by the factors, defined as the sum of squared factor loadings for each variable).

### Age decade comparisons of lipid abundances

**Fig 3:**
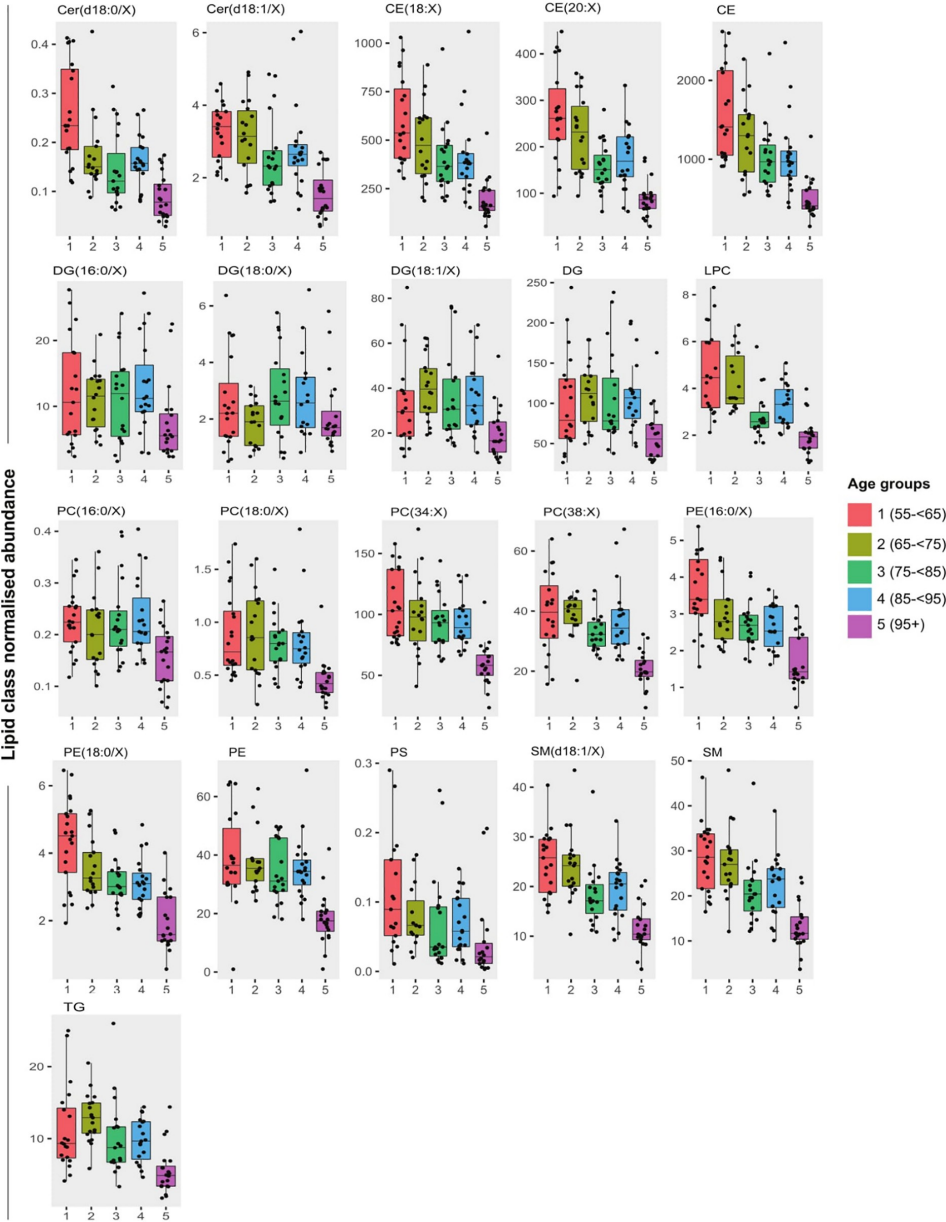
Boxplots of lipid class normalised abundance across age groups. The 95+ group had significantly lower abundance compared to younger age groups (Kruskall Wallis test and pairwise Mann Whitney U-tests, p<0.05) for all lipid classes with the exception of DG lipids and subclasses.

Upon comparing abundances by age group, subjects from the oldest age decade (subjects from the SCS aged 95+) had significantly lower lipids for all lipid classes examined relative to all other age groups (Fig 3, p<0.05, Kruskall Wallis test and *post-hoc* pairwise Mann-Whitney U tests), with the exception of DG lipids and their subclasses. Negative relationships of lipids by raw age are also shown in S1 Fig, and in S4 Table.

### Linear regression

Multiple linear regressions were applied to model lipid abundances using age centred at 75 years of age (age c75) as independent variables and BMI, sex and the interaction of age with sex as control variables (Table 3). Since the distributions of lipid abundances were found to be positively skewed, they were first log_10_ transformed to more closely approximate the normal distribution. All lipid classes were significantly and negatively affected by age from 56–100 years (p<0.05), with the exception of DG(18:0/X) (p<0.10), while DG(18:1/X) did not yield a significant model (p>0.05).

**Table 3:** Regression models of log-10 transformed lipid abundances as a function of age, BMI, sex and age by sex interaction.

Plasma Lipid Category		B Agec75	B Agec75 F^a^	B BMI	B Sex	B Interaction Sex × Agec75	R Squared
Cer(d 18:0/X)		-0.013***	-0.008***	-0.002	0.019	0.007**	0.32***
Cer(d 18:1/X)		-0.009***	-0.005**	-0.005	0.038	0.005^†^	0.25***
CE(18:X)		-0.014***	-0.011***	0.007^†^	-0.003	0.003	0.47***
CE(20:X)		-0.015***	-0.01***	0.009**	-0.005	0.006**	0.47***
CE		-0.014***	-0.011***	0.008**	-0.001	0.004	0.47***
DG(16:0/X)		-0.012***	0.002	0.003	-0.08	0.014**	0.09*
DG(18:0/X)		-0.007^†^	0.007*	0.004	-0.027	0.014**	0.06*
DG(18:1/X)		-0.009**	-0.001	0.001	-0.044	0.008^†^	0.05^†^
DG		-0.01***	0.001	0.002	-0.046	0.01**	0.08*
LPC		-0.011***	-0.009***	-0.007**	-0.006	0.002	0.39***
PC(16:0/X)		-0.005**	-0.001	-0.004	0.002	0.005**	0.06*
PC(18:0/X)		-0.01***	-0.003^†^	0.002	-0.002	0.007**	0.16***
PC(34:X)		-0.008***	-0.004*	-0.001	-0.008	0.004**	0.21***
PC(38:X)		-0.009***	-0.003*	0.001	-0.001	0.006**	0.30***
PE(16:0/X)		-0.011***	-0.005**	-0.002	0.01	0.006**	0.35***
PE(18:0/X)		-0.011***	-0.004*	-0.001	0.026	0.006**	0.36***
PE		-0.011***	-0.007***	0.002	-0.03	0.004^†^	0.37***
PS		-0.02***	-0.01^†^	-0.005	0.011	0.009	0.11***
SM(d 18:1/X)		-0.007***	-0.008***	-0.002	0.04	0.001	0.33***
SM		-0.007***	-0.007***	-0.001	0.037	-0.00003	0.33***
TG		-0.012***	-0.004^†^	0.003	-0.014	0.007**	0.21***
	**Principal Component Analysis Exploratory Factors**						
FAC1		-0.066***	-0.042***	-0.009	0.053	0.024**	0.55***
FAC2		-0.042***	0.007	0.016	-0.098	0.049**	0.14***

Plasma lipids were log10-transformed to reduce skewness of dependent variables. B represents unstandardized regression coefficients. Agec75 is age centred at 75 years (i.e. actual age– 75 years). R squared is the adjusted R squared value. Interaction refers to the product term Agec75*Sex. Factor scores (FAC1 and FAC2) were derived from principal component analysis of lipid class variables. Sex was coded male = 0; female = 1. Hence positive regression coefficients for the interaction terms indicate stronger negative effects of age for males.

^a^ Regression coefficients were also calculated separately for females by recoding female = 0, male = 1. Thus B Agec75 F represents the effect of age on lipids in females only, compared against B Agec75 which represents the effect of age on lipids in males only.

^†^ p<0.10

*p < .05

**p < .01

***p < .001.

BMI was significantly associated with increased CE (p<0.05, and p<0.10 for CE(18:X)), and decreased LPC (p<0.05), though there were no overall no statistically significant relationships with the other lipid classes. None of regression coefficients representing the main effects of sex were statistically significant (note that, since a sex by age interaction term was included in the model, these regression coefficients represent the effects of sex at the centred value of age, namely at age 75). To assess sex differences in the relationship between age and lipid classes, we included a product term to represent the interaction of age with sex. While sex was not significant for all classes at age 75, there were significant positive interactions of age with sex for Cer, DG, TG, PC and PE subclasses, as well as CE(20:X), suggesting the negative effect of age was stronger in males (p<0.05). In addition, we determined sex-specific effects of age on the lipidome by recoding the sex term (swapping males and females) to isolate regression coefficients of age for females only (Table 3). This confirmed stronger negative effects of age on lipids in males compared to females for most lipid classes. In general, the combined effect of all variables in the equation explained from 6% up to 47% of the variance in each model.

We also examined as dependent variables the two sets of component scores the two factors derived from principal component analyses. The overall models were significant, with component scores FAC1 and FAC2 both significantly and negatively associated with age, and with positive and statistically significant age-sex interaction, with the predictor variables in combination explaining 56% and 14% of the total variance of FAC1 and FAC2, respectively.

### Associations of sex with lipid classes

**Fig 4:**
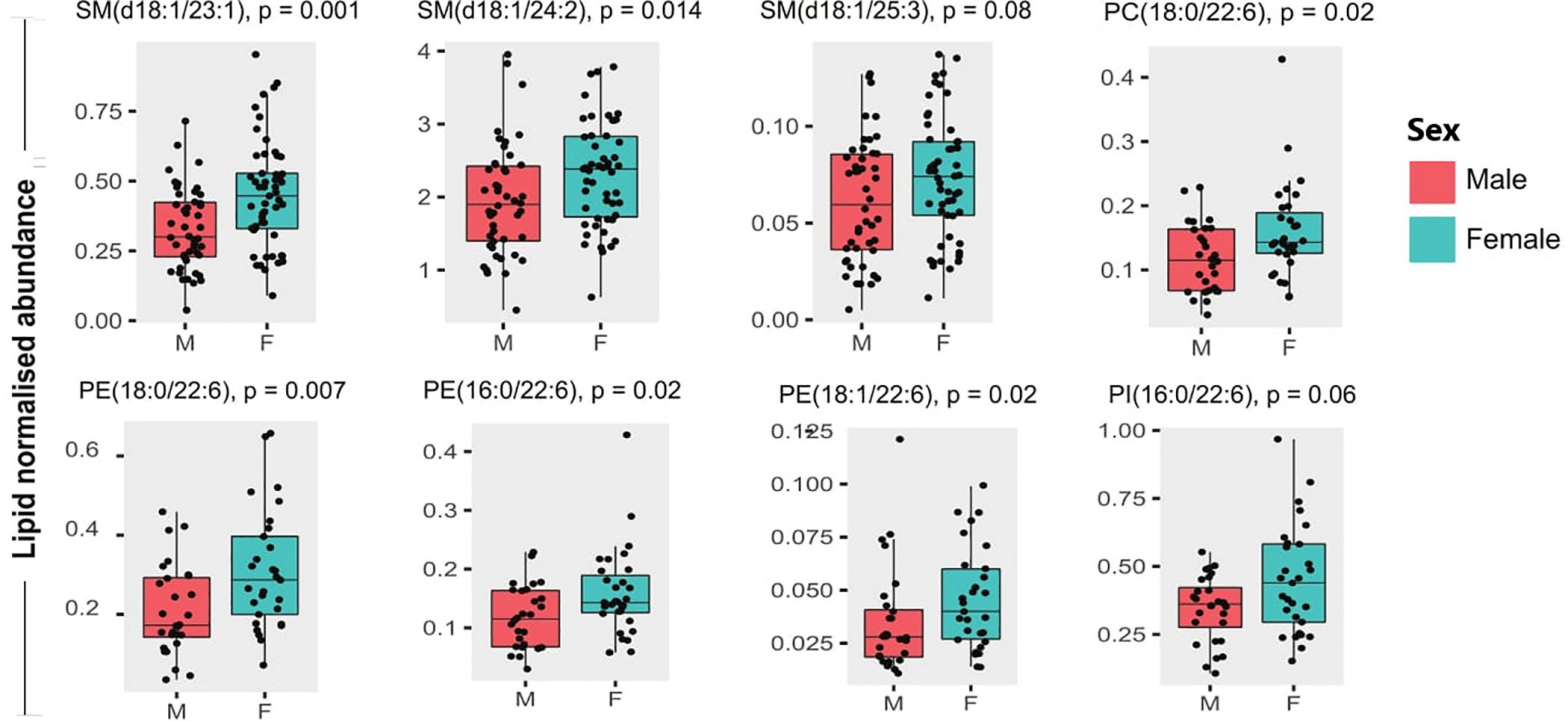
Boxplots of lipid normalised abundances for specific sphingomyelins and phospholipids. Note phospholipids presented were taken from subjects aged over 75 years by sex; p-values derived based on Mann-Whitney U test.

Our regression model (Table 3) did not find independent sex differences between lipid classes (all p>0.05). However, we did find individual species of SM were higher in females than in males (Fig 4, p<0.05, Mann-Whitney U test). In particular, SM(d18:1/24:2) and SM(d18:1/23:1) were higher in females than males, and SM(d18:1/25:3) trended higher in females (p = 0.07). Among subjects aged over 75 years, females also had higher levels of DHA–containing phospholipids including PE(18:0/22:6) and PC(18:0/22:6) in positive mode, and PE(18:1/22:6), and PE(16:0/22:6) in negative mode, with PI(16:0/22:6) trending higher but not reaching significance (Fig 4, p = 0.06).

Additionally, among females, PC(18:0/22:6), PE(16:0/22:6), PE(18:0/22:6) and PE(18:1/22:6) correlated with enzymatically assayed TG levels (r = 0.48, 0.48, 0.59 and 0.43 respectively, p<0.01), while PE(16:0/22:6), PE(18:0/22:6) and PE(18:1/22:6) were also negatively correlated with LDL-C levels (r = -0.37, -0.35, and -0.46 respectively, p<0.02) independent of age and BMI.

## Discussion

Our study examined how plasma lipids are differentially associated with ageing, sex and BMI during the second half of the human lifespan in *APOE* ε*3* homozygous individuals. Our untargeted LC-MS experiment, using a cross-sectional approach, detected a general decline in plasma lipid metabolites among older participants, especially in the oldest subjects aged between 95 to 100 years. Furthermore, we found some sex specific differences in lipid profiles, particularly in levels of specific SMs and DHA-containing phospholipids. Surprisingly, few associations of lipid level and BMI were identified.

Untargeted metabolomics approaches have commonly been used to identify relative changes in the plasma lipidome in health and disease [2, 8]. Previous studies have identified associations of plasma lipid profiles with age, in age brackets up to about 65 years of age. For example, one study focusing on individuals aged between 20–65 years [12], found that the concentrations of 100 metabolites are differentially altered with age, with fewer lipids noted to differ by sex and race. Selected lipid metabolites, including fatty acids and cholesterol were increased in concentration in subjects aged between 51–65 years of age compared to younger subjects. The study also reported higher levels of lipid metabolites in females compared to males, independent of age, indicating potential sex related differences. Another study described changes in concentrations of plasma lipids between two age groups, a young (25–35 years) and an old (55–65) age group [8]. In males and females, eight and 89 lipids, respectively, showed significant associations with age. Thus, the study indicates that a majority of lipids remain stable with age, especially in males, while some lipids may be increased from young through to middle age.

### Age–specific differences in lipid profiles

There is a major point of difference between the present study and previous work, in that we focused on individuals from 56 up to 100 years of age (mean = 77.8), while most published work report a maximum age of 65. Herein, we have investigated the plasma lipidome during the second half of the human lifespan, including a cohort of subjects aged 95 and above enrolled in the SCS [17]. These extreme aged subjects are important as they represent a model of successful ageing [25], typically surviving 20 years longer than the average life expectancy. The mechanism(s) which confer this extreme longevity are unclear, and possibilities include *(i)* lower incidences of serious diseases and risk of morbidity compared to younger cohorts [25–27], *(ii)* a surplus of protective mechanisms which either circumvent the impact of stressors or else confer resilience in spite of disease or the impact of stressors or *(iii)* “compression of morbidity” [26]. Investigating the lipidome of aged and super-aged individuals may identify indices related to healthy ageing, where certain blood-borne factors may be implicated in longevity [28], especially as lipids have previously been linked to age-related disease and brain ageing [2, 6, 29]. Furthermore, there is evidence that centenarians are likely to have unique and heritable genetic makeup that predisposes them to a longer life [30, 31]. These genetic protective factors may manifest in altered lipid composition or lipoprotein particle size [32], although modifiable lifestyle factors, such as diet, also play a role in longevity and regulation of lipid homeostasis [30].

The results of our study suggest that there is a universal decline in the plasma concentration of all major lipid classes during the second half of the human lifespan in healthy individuals. In particular, the concentration of plasma lipids was lowest in the 95+ age group, irrespective of lipid class. Interestingly, this particular group of extreme aged individuals were largely taken from the SCS and markedly reduced lipid concentrations compared to all other (younger) age groups, which we further confirmed using linear regression models by age. In contrast with previous studies which compare young adults to middle aged adults, where lipids are generally reported to be increased with age [8, 12], in our cohort of much older individuals, the trend is to a plateau or slight lowering of plasma lipid levels in middle to older age, and a marked drop in the centenarian age bracket. Our data suggest that the lipidome in ‘oldest-old’ people could differ substantially to that observed in middle-aged adults, possibly with a reversal to patterns seen among younger subjects [33], though data in younger subjects is required for direct comparison.

Various studies have indicated a lipidomic signature predictive of longevity [33, 34]. For example, longevity has been specifically associated with reduced concentrations of plasma long chain free fatty acids in mammals, especially polyunsaturated fatty acids. A distinct lipidomic signature for ageing has also been identified in humans, where a lower double bond index, or reduced lipid peroxidisability of fatty acids was found to correlate with increasing longevity [35]. These differences were only noted in subjects aged over 95 years, and not in elderly subjects with a mean age of 75 years [33], thus necessitating the study of the human lipidome into extreme age to fully uncover these age specific effects. Additionally, it is important to examine functional consequences of lipid and lipoprotein modulating gene variants, which have previously been identified in genomic studies [36].

Other studies comparing centenarians against elderly subjects (mean age = 70 yrs) from Northern Italy [7, 34] found SM to be elevated in centenarians, while some phospholipids were also different between groups. In contrast, we found an overall reduction of SM species with extreme ageing. These differences could be related to the fact that the centenarians sampled in the Montoliu study [7] focused largely on females, in which we found SM and LDL-C levels to be elevated compared to males. Secondly, the data is derived from an ageing population in Northern Italy, where dietary, lifestyle and genetic influences could be substantially different from that of the typical older Australian recruited in the SCS [17]. Also, we stratified for *APOE* ε*3* homozygous individuals while it is unlikely this study selected for *APOE* genotype. However, the Montoliu study did find that most other phosphocholines were significantly decreased in the elderly [7], which mirrors our overall findings.

We further showed that plasma lipid levels may be predicted by a linear combination of age, BMI, sex and the interaction of age and sex, and this is lipid class dependent. In particular, all lipids apart from DG(18:0/X) covaried with age. DG lipids had the lowest variance explained and this may be a result of an inverted U-shaped trajectory across age (Fig 2). Although the sex term was not significant for any lipid class, significant interactions of age with sex for Cer, phospholipids, DG and TG indicate that some lipid classes could decline with age differently between the sexes, with a steeper decline among males, also confirmed by the more negative regression coefficients for age in males compared to females. We were also able to factor-analyse lipid classes using principal component analysis to identify two factors explaining most of the variance, which are likely related to LDL or ApoB, and TG-rich VLDL. As with individual lipid classes, both factors when entered into our linear regression model were significantly and negatively associated with age, as well as age-sex interaction, such that females had higher levels of lipids compared to males. The results are consistent with previous findings from a study in individuals aged from 50–55 years where females had increased levels of ApoB to overtake that of males. Previously, our laboratory also showed that apolipoprotein expression is altered in older individuals, with most plasma apolipoproteins being reduced in older subjects, but are also higher among females compared to males over the age of 55 [37]. Therefore, it is possible that the decline in plasma lipids could be related to marked changes in circulating lipid transporters and carrier proteins in older age. Other mechanisms such as altered hepatic lipid metabolism, expression of LDL receptors, and intestinal uptake of lipids is known to affect plasma lipid profiles in middle aged adults under the age of 60 [38–40], though this may be different in older aged adults.

### Impact of lipid-lowering medication on lipid profiles

One potential confounder is that some of the subjects taken from the SCS were also on lipid-lowering medication. Our analysis revealed that subjects on lipid-lowering medication had reduced LDL-C and total cholesterol, and increased HDL-C. Nevertheless, even after excluding these subjects from analysis, the negative association of lipid levels with age was preserved. In fact, there were more subjects on lipid-lowering medications in the 85-<95 group compared to the 95+ group (n = 8 vs n = 5 respectively). We note that many ageing studies fail to report the use of lipid-lowering medication in their subjects and the potential effect this might have on lipid profiles. In any case, our data suggest that while lipid-lowering medication can alter lipid profiles, namely LDL-C, HDL-C and total cholesterol, an age-related reduction in plasma lipids may be independent of these effects, though only a small number of subjects were on lipid-lowering medication in our study.

### Sex–specific differences in lipid profiles

Previous studies in younger subjects have shown substantial sex differences in the plasma lipidome [8, 13]. Our results confirm that some sex differences hold up even in this older age range. Of note, we found significant sex differences in the levels of particular lipids in the SM, PC and PE families. In particular, SM(d18:1/23:1) and SM(d18:1/24:2) were elevated in females, which has previously been reported [8, 41] and could potentially be accounted for by differences in LDL-C cholesterol levels. We found that elderly females have higher levels of LDL-C, HDL-C and total cholesterol compared to elderly males and this could be linked to increased levels of apoA-I and apoB in females [37, 42]. However, there is some variance in the literature on this topic, since another study found that males had higher LDL-C levels compared to females in subjects aged under 55 years, although among females, LDL-C and apoB levels increased post-menopause [42, 43]. Additionally, it has been suggested that physical activity could confer greater benefits towards increasing HDL-C levels among middle aged and elderly females [44] compared to similarly aged males. If so, this necessitates differential approaches to treatment and prevention of dyslipidaemia and atherosclerosis between sexes, such as lipidomic screening of postmenopausal women [43].

DHA-containing fatty acids in plasma phospholipids, such as PE(18:0/22:6), PE(16:0/22:6) and PE(18:1/22:6) and PC(18:0/22:6), were also reduced in males aged over 75 years, relative to females, an observation also reported in previous studies [8, 45]. This is potentially attributable to differences in liver expression of enzymes involved in DHA biosynthesis, particularly desaturases and elongases [46]. Whether this confers a difference in longevity between sexes is still unclear, though we were able to show a negative correlation between some of these phospholipids with triglyceride and LDL-C levels among females, which could be indicative of an anti-atherogenic profile in extreme aged females. Additionally, DHA is considered vital in maintaining brain health and cognition [47], and higher levels of phospholipid DHA has been shown to be associated with decreased mortality due to late-life disease [48].

In young females, levels of sphingolipids and cholesterol are increased from young age to middle age, and this effect is elevated when contraceptives are used [49], suggesting a strong interplay between sex hormones and lipid levels. Given that our cohort features solely post-menopausal women, lipid differences by sex appear to persist in older age. Previous studies have reported that postmenopausal women typically have a more pro-atherogenic lipid profile compared to pre-menopausal women [49, 50]. There were no other sex differences in lipid levels apart from LDL-C, HDL-C, total cholesterol, various species of SM and DHA-fatty acid containing PL levels, although we did note that the decline of lipid levels with increasing age may be different between sexes. In particular, the significant interaction of age with sex and stronger negative regression coefficients of age in males in our regression models indicate that males tend to show a steeper rate of decline in lipids (especially phospholipids) with age compared to females. While the mechanism is unclear, it could be related to inherent differences in metabolism between sexes, where males tend to oxidise lipids faster than females [51], as well as the effects of menopause and hormone replacement therapy [52]. Genetic and epigenetic factors could also be involved, as some lipids are associated with familial longevity among female offspring of nonagerians [53]. Overall, our results indicate plasma lipidomic changes with age is modifiable by sex in healthy individuals, and it is likely that sex also impacts on the lipidome in age-related diseases such as Alzheimer’s disease, modifying risk and severity [54–57].

### BMI association with lipids

One surprising finding was the minimal association between BMI with most lipid classes studied. While high BMI is typically associated with obesity and related conditions such as diabetes and cardiovascular disease, the exact relationship between BMI and lipid levels in ‘healthy’ individuals remains unclear. Some studies have shown a lack of association between LDL-C and higher BMI. For example, Manjareeka, Nanda [58] found that BMI had a very weak association with LDL-C, HDL-C and total cholesterol [58], with no discernible differences between normal (BMI = 20–25 kg/m^2^) and overweight or obese subjects (BMI>25 kg/m^2^). The authors suggested that BMI may not be an accurate indicator of fat mass since measured weight does not discriminate between lean and fat mass. BMI also does not take into account intrinsic differences in fat mass between sexes, and at different ages [59, 60]. In particular, BMI tends to overestimate fat mass in males and underestimate fat mass among the elderly, which could complicate our understanding of our results in relation to the elderly. Another study comparing monozygotic twins with notable BMI differences of at least 4 kg/m^2^ found that obesity is associated with increases in lysophosphatidylcholines, while some other lipid classes, such as SM, remained largely unchanged among twin pairs [15]. A similar BMI difference is defined in our groups (18–25 vs 28+) but we found surprisingly few associations of BMI with lipid class. In particular, we did not find any differences between BMI groups (<25 vs 28+) and regression analysis only showed a significant association of BMI with increased CE levels and reduced LPC levels after controlling for age and sex. The findings regarding LPC levels contradicts the above study, but there is variance in the literature regarding the LPC and obesity, where other studies suggest lower LPC levels is correlated with obesity, insulin resistance and BMI [61, 62]. The apparent contradiction may be due to the vast age differences in the various studies, with the study of twins undertaken in young subjects under the age of 30 years, while other studies of obesity and type II diabetes recruited middle-aged subjects up to the age of 60 years, which is an age range closer to that of our study. Our study is one of the first lipidomics studies of BMI in older aged individuals, and we found minimal associations of lipids with BMI, apart from an increase in CE and a reduction in LPC.

### Limitations

Although our study provides a good overview of lipidomic changes that are associated with age, sex and BMI in older individuals, there are also some limitations: *(i)* we focused on subjects aged over 55 years, which prevents direct comparisons with younger and middle aged adults; *(ii)* a largely Caucasian population living in South-eastern Australia was used, so caution must be taken when interpreting results against that of studies in non-Caucasian subjects, where racial differences may be evident [44]; *(iii)* our sample size of 100 subjects, split by sex, is modest from a statistical standpoint (though similar or even larger than some studies reported in the literature). Ideally, a larger range of subjects in the order of several hundred subjects should be used to increase power; and *(iv)* most studies, including the present study, use cross-sectional designs and may therefore be influenced by cohort effects. We suggest that further studies should be conducted in a longitudinal setting to fully understand intra-individual variation in lipids with age, although longitudinal studies over several decades are generally impractical unless historical samples are available.

The question then remains as to the potential mechanism behind the global decline in lipids noted in subjects from the SCS cohort. Our study is limited in that we did not include subjects over the age of 95 from any other cohorts, and thus it is difficult to ascertain whether the sudden decrease in lipids noted in the 95+ group could potentially be a specific cohort effect rather than an age effect. Even so, all participants were sampled from Eastern Australia with similar socio-economic backgrounds and we attempted to control for other patient variables as much as possible so that they were similar between cohorts (S2 Table). Furthermore, the blood collection process performed is routine for all studies and analysis of samples was performed at wave 1. We previously used the same cohorts with a larger number of subjects to quantify apolipoproteins among participants aged from 55–100 and found changes in most apolipoproteins with age consistent with the lipidomics data gathered here [37]. Our results indicate that the SCS is a unique cohort with a significant reduction in lipid levels likely a result of advanced age [26], but further work is required to verify this.

Another limitation is that we largely used total lipids within a class/subclass in order to simplify analysis, with arbitrary grouping according to total number of carbons or double bonds. Nevertheless, we uncovered a great deal of redundancy in that many individual lipid species and subclasses of a single class behaved similarly with respect to age and sex, and were also largely inter-correlated. This suggests that summing lipids as a group could be a simple and efficient way to analyse global lipid changes, although it is important to note particular species of lipids within a class may have distinct functions, and could be differentially implicated for some conditions. Indeed, although we found total SM(d18:1/X) was not significantly different between males and females across age, some individual SM(d18:1/X) species were different based on sex. Our study covered a large range of different lipid classes found in plasma, though further coverage of the lipidome is possible through other analytical techniques, such as nuclear magnetic resonance spectroscopy (NMR) [7, 34], and GC-MS [12, 33, 35]. Finally, we emphasise that as this is a basic research study (and not an epidemiological study) as the subject group was comprised of healthy individuals and we removed the *APOE* allele confounder by selecting individuals with the *APOE E3/E3* genotype. We acknowledge that this approach could have a bearing on the abundance of lipids uncovered, especially as the *APOE* genotype is a common risk factor for age related diseases such as Alzheimer’s disease [10] and late life cognitive decline [21, 37, 56]. Even so, we expect our results to be largely reflective of ageing in the general eastern Australian population, with >70% being *APOE* ε*3* homozygous, and furthermore, the prevalence of *APOE E3/E3* genotype is typically enhanced in older subjects relative to *APOE* ε*4* carriers [63]. In addition, focusing on a single and common *APOE* genotype increases the power of our study where we would otherwise have to statistically control for this variable. In future work, we intend to survey the impact of *APOE* genotypes on lipid profiles.

## Conclusions

Our untargeted lipidomics approach using plasma from 100 healthy subjects aged from 56 to 100 indicates a universal reduction in lipid profiles in older subjects independent of sex and BMI, which is especially apparent in the oldest subjects over 95 years of age. Our study also suggests that the lipid profile with age is substantially different in the elderly population compared to previous reports in young and middle aged adults. There were fewer sex related differences in lipids, which included higher levels of specific SMs and DHA-containing phospholipids, as well as LDL-C, HDL-C and total cholesterol among females. Also, the reduction of most lipids in older age was strongest among males. BMI was only associated with increased CE and reduced LPC. Results of our small study highlight the importance of understanding and accounting for natural variation in the plasma lipidome in experimental designs. Further studies in larger independent cohorts are recommended to clarify and validate these findings, which could highlight important processes involved in healthy ageing and longevity.

## Supporting information

S1 Appendix Supporting methods. (DOCX)

S1 Table Lipid classes and number of lipids analysed in positive and negative ion mode. (DOCX)

S2 Table Patient characteristics and lipid profiles by age cohort. (DOCX)

S3 Table Patient characteristics and lipid profiles by sex. (DOCX)

S4 Table Correlations of lipid class normalised abundances with age. Correlations were taken for all subjects, then after correcting for sex, BMI and lipid-lowering medication usage, and after excluding those on lipid-lowering medication.
(DOCX)

S1 Fig Scatterplot of age (years) with normalised lipid abundances for each lipid category. (DOCX)

## References

[1] Brugger B., Lipidomics: analysis of the lipid composition of cells and subcellular organelles by electrospray ionization mass spectrometry. Annu Rev Biochem, 2014 83: p. 79–98. 10.1146/annurev-biochem-060713-035324 24606142

[2] Meikle P.J., et al, Lipidomics: Potential role in risk prediction and therapeutic monitoring for diabetes and cardiovascular disease. Pharmacology & Therapeutics, 2014 143(1): p. 12–23. 24509229 10.1016/j.pharmthera.2014.02.001

[3] Zuo H., et al, Plasma Biomarkers of Inflammation, the Kynurenine Pathway, and Risks of All-Cause, Cancer, and Cardiovascular Disease Mortality: The Hordaland Health Study. Am J Epidemiol, 2016 183(4): p. 249–58. 10.1093/aje/kwv242 26823439 PMC4753283

[4] Gonzalez-Dominguez R., Garcia-Barrera T., and Gomez-Ariza J.L., Combination of metabolomic and phospholipid-profiling approaches for the study of Alzheimer's disease. J Proteomics, 2014 104: p. 37–47. 10.1016/j.jprot.2014.01.014 24473279

[5] Wong M.W., et al, The application of lipidomics to biomarker research and pathomechanisms in Alzheimer's disease. Curr Opin Psychiatry, 2017 30(2): p. 136–144. 10.1097/YCO.0000000000000303 28002106

[6] Wong M.W., et al, Dysregulation of lipids in Alzheimer's disease and their role as potential biomarkers. Alzheimer's & Dementia: The Journal of the Alzheimer's Association, 2017 13(7): p. 810–827. 10.1016/j.jalz.2017.01.008 28242299

[7] Montoliu I., et al, Serum profiling of healthy aging identifies phospho- and sphingolipid species as markers of human longevity. Aging (Albany NY), 2014 6(1): p. 9–25. 10.18632/aging.100630 24457528 PMC3927806

[8] Ishikawa M., et al, Plasma and serum lipidomics of healthy white adults shows characteristic profiles by subjects' gender and age. PLoS One, 2014 9(3): p. e91806 10.1371/journal.pone.0091806 24632803 PMC3954792

[9] Alshehry Z.H., et al, An Efficient Single Phase Method for the Extraction of Plasma Lipids. Metabolites, 2015 5(2): p. 389–403. 10.3390/metabo5020389 26090945 PMC4495379

[10] Bandaru V.V., et al, ApoE4 disrupts sterol and sphingolipid metabolism in Alzheimer's but not normal brain. Neurobiol Aging, 2009 30(4): p. 591–9. 10.1016/j.neurobiolaging.2007.07.024 17888544 PMC2758772

[11] Ferreira C.N., et al, Comparative study of apolipoprotein-E polymorphism and plasma lipid levels in dyslipidemic and asymptomatic subjects, and their implication in cardio/cerebro-vascular disorders. Neurochem Int, 2010 56(1): p. 177–82. 10.1016/j.neuint.2009.09.016 19819279

[12] Lawton K.A., et al, Analysis of the adult human plasma metabolome. Pharmacogenomics, 2008 9(4): p. 383–97. 10.2217/14622416.9.4.383 18384253

[13] Sales S., et al, Gender, Contraceptives and Individual Metabolic Predisposition Shape a Healthy Plasma Lipidome. 2016 **6**: p. 27710 10.1038/srep27710 27295977 PMC4906355

[14] Rauschert S., et al, Lipidomics Reveals Associations of Phospholipids With Obesity and Insulin Resistance in Young Adults. J Clin Endocrinol Metab, 2016 101(3): p. 871–9. 10.1210/jc.2015-3525 26709969

[15] Pietiläinen K.H., et al, Acquired Obesity Is Associated with Changes in the Serum Lipidomic Profile Independent of Genetic Effects–A Monozygotic Twin Study. PLOS ONE, 2007 2(2): p. e218 10.1371/journal.pone.0000218 17299598 PMC1789242

[16] Sachdev P.S., et al, The Sydney Memory and Ageing Study (MAS): methodology and baseline medical and neuropsychiatric characteristics of an elderly epidemiological non-demented cohort of Australians aged 70–90 years. International Psychogeriatrics, 2010 22(8): p. 1248–1264. 10.1017/S1041610210001067 20637138

[17] Sachdev P.S., et al, The Sydney Centenarian Study: methodology and profile of centenarians and near-centenarians. Int Psychogeriatr, 2013 25(6): p. 993–1005. 10.1017/S1041610213000197 23510643

[18] McEvoy M., et al, Cohort profile: The Hunter Community Study. Int J Epidemiol, 2010 39(6): p. 1452–63. 10.1093/ije/dyp343 20056765

[19] Dufouil C., et al, APOE genotype, cholesterol level, lipid-lowering treatment, and dementia: the Three-City Study. Neurology, 2005 64(9): p. 1531–8. 10.1212/01.WNL.0000160114.42643.31 15883313

[20] Hyotylainen T. and Oresic M., Optimizing the lipidomics workflow for clinical studies—practical considerations. Anal Bioanal Chem, 2015 407(17): p. 4973–93. 10.1007/s00216-015-8633-2 25855150

[21] Song F., et al, Plasma Apolipoprotein Levels Are Associated with Cognitive Status and Decline in a Community Cohort of Older Individuals. PLoS ONE, 2012 7(6): p. e34078 10.1371/journal.pone.0034078 22701550 PMC3372509

[22] Wang M., Wang C., and Han X., Selection of internal standards for accurate quantification of complex lipid species in biological extracts by electrospray ionization mass spectrometry-What, how and why? Mass Spectrom Rev, 2016. 10.1002/mas.21492 PMC4947032 26773411

[23] Benjamini Y. and Hochberg Y., Controlling the False Discovery Rate: A Practical and Powerful Approach to Multiple Testing. Journal of the Royal Statistical Society. Series B (Methodological), 1995 57(1): p. 289–300.

[24] Fahy E., et al, Update of the LIPID MAPS comprehensive classification system for lipids. J Lipid Res, 2009 50 **Suppl**: p. S9–14. 10.1194/jlr.R800095-JLR200 19098281 PMC2674711

[25] Kheirbek R.E., et al, Characteristics and Incidence of Chronic Illness in Community-Dwelling Predominantly Male U.S. Veteran Centenarians. Journal of the American Geriatrics Society, 2017: p. n/a-n/a. 10.1111/jgs.14900 PMC5724032 28422270

[26] Andersen S.L., et al, Health span approximates life span among many supercentenarians: compression of morbidity at the approximate limit of life span. J Gerontol A Biol Sci Med Sci, 2012 67(4): p. 395–405. 10.1093/gerona/glr223 22219514 PMC3309876

[27] Barzilai N., et al, Unique lipoprotein phenotype and genotype associated with exceptional longevity. Jama, 2003 290(15): p. 2030–40. 10.1001/jama.290.15.2030 14559957

[28] Castellano J.M., Kirby E.D., and Wyss-Coray T., Blood-Borne Revitalization of the Aged Brain. JAMA Neurol, 2015 72(10): p. 1191–4. 10.1001/jamaneurol.2015.1616 26237737 PMC4867550

[29] Ledesma M.D., Martin M.G., and Dotti C.G., Lipid changes in the aged brain: Effect on synaptic function and neuronal survival. Progress in Lipid Research, 2012 51(1): p. 23–35. 10.1016/j.plipres.2011.11.004 22142854

[30] Puca A.A., Chatgilialoglu C., and Ferreri C., Lipid metabolism and diet: Possible mechanisms of slow aging. The International Journal of Biochemistry & Cell Biology, 2008 40(3): p. 324–333. 17509925 10.1016/j.biocel.2007.04.003

[31] Borras C., et al, Human exceptional longevity: transcriptome from centenarians is distinct from septuagenarians and reveals a role of Bcl-xL in successful aging. Aging (Albany NY), 2016 8(12): p. 3185–3201. 27794564 10.18632/aging.101078 PMC5270663

[32] Milman S., et al, Phenotypes and Genotypes of High Density Lipoprotein Cholesterol in Exceptional Longevity. Current vascular pharmacology, 2014 12(5): p. 690–697. 24350928 10.2174/1570161111666131219101551 PMC4087084

[33] Jove M., et al, A Stress-Resistant Lipidomic Signature Confers Extreme Longevity to Humans. J Gerontol A Biol Sci Med Sci, 2016. 10.1093/gerona/glw048 27013396

[34] Collino S., et al, Metabolic Signatures of Extreme Longevity in Northern Italian Centenarians Reveal a Complex Remodeling of Lipids, Amino Acids, and Gut Microbiota Metabolism. PLOS ONE, 2013 8(3): p. e56564 10.1371/journal.pone.0056564 PMC3590212 23483888

[35] Jové M., et al, Plasma long-chain free fatty acids predict mammalian longevity. Scientific Reports, 2013 **3**: p. 3346 10.1038/srep03346 24284984 PMC3842621

[36] Dron J.S. and Hegele R.A., Genetics of Lipid and Lipoprotein Disorders and Traits. Current Genetic Medicine Reports, 2016 4(3): p. 130–141. 10.1007/s40142-016-0097-y 28286704 PMC5325854

[37] Muenchhoff J., et al, Plasma apolipoproteins and physical and cognitive health in very old individuals. Neurobiol Aging, 2017 55: p. 49–60. 10.1016/j.neurobiolaging.2017.02.017 28419892

[38] Woudstra T.D., et al, The age-related decline in intestinal lipid uptake is associated with a reduced abundance of fatty acid-binding protein. Lipids, 2004 39(7): p. 603–10. 15588016 10.1007/s11745-004-1272-9

[39] Field P.A. and Gibbons G.F., Decreased hepatic expression of the low-density lipoprotein (LDL) receptor and LDL receptor-related protein in aging rats is associated with delayed clearance of chylomicrons from the circulation. Metabolism, 2000 49(4): p. 492–8. 10778874 10.1016/s0026-0495(00)80014-1

[40] Mc Auley M.T. and Mooney K.M., Computationally Modeling Lipid Metabolism and Aging: A Mini-review. Computational and Structural Biotechnology Journal, 2015 13: p. 38–46. 10.1016/j.csbj.2014.11.006 25750699 PMC4348429

[41] Mielke M.M., et al, Factors affecting longitudinal trajectories of plasma sphingomyelins: the Baltimore Longitudinal Study of Aging. Aging Cell, 2015 14(1): p. 112–21. 10.1111/acel.12275 25345489 PMC4310757

[42] Anagnostis P., et al, Effects of gender, age and menopausal status on serum apolipoprotein concentrations. Clin Endocrinol (Oxf), 2016 85(5): p. 733–740. 10.1111/cen.13085 27086565

[43] Goh V.H., et al, Differential impact of aging and gender on lipid and lipoprotein profiles in a cohort of healthy Chinese Singaporeans. Asian J Androl, 2007 9(6): p. 787–94. 10.1111/j.1745-7262.2007.00294.x 17968464

[44] Monda K.L., Ballantyne C.M., and North K.E., Longitudinal impact of physical activity on lipid profiles in middle-aged adults: the Atherosclerosis Risk in Communities Study. Journal of Lipid Research, 2009 50(8): p. 1685–1691. 10.1194/jlr.P900029-JLR200 19346332 PMC2724055

[45] Lohner S., et al, Gender Differences in the Long-Chain Polyunsaturated Fatty Acid Status: Systematic Review of 51 Publications. Annals of Nutrition and Metabolism, 2013 62(2): p. 98–112. 10.1159/000345599 23327902

[46] Kitson A.P., et al, Tissue-specific sex differences in docosahexaenoic acid and Δ6-desaturase in rats fed a standard chow diet. Applied Physiology, Nutrition, and Metabolism, 2012 37(6): p. 1200–1211. 10.1139/h2012-103 23050796

[47] Weiser M.J., Butt C.M., and Mohajeri M.H., Docosahexaenoic Acid and Cognition throughout the Lifespan. Nutrients, 2016 8(2): p. 99 10.3390/nu8020099 26901223 PMC4772061

[48] Mozaffarian D., et al, Plasma phospholipid long-chain omega-3 fatty acids and total and cause-specific mortality in older adults: a cohort study. Ann Intern Med, 2013 158(7): p. 515–25. 10.7326/0003-4819-158-7-201304020-00003 23546563 PMC3698844

[49] Anagnostis P., et al, Effects of menopause, gender and age on lipids and high-density lipoprotein cholesterol subfractions. Maturitas, 2015 81(1): p. 62–8. 10.1016/j.maturitas.2015.02.262 25804951

[50] Saha K.R., et al, Changes in lipid profile of postmenopausal women. Mymensingh Med J, 2013 22(4): p. 706–11. 24292300

[51] Varlamov O., Bethea C.L., and Roberts C.T. Jr., Sex-specific differences in lipid and glucose metabolism. Front Endocrinol (Lausanne), 2014 5: p. 241. 25646091 10.3389/fendo.2014.00241 PMC4298229

[52] Ki E.Y., et al, Differences in the lipid profile and hormone replacement therapy use in Korean postmenopausal women: the Korea National Health and Nutrition Examination Survey (KNHANES) 2010–2012. Archives of Gynecology and Obstetrics, 2016 294: p. 165–173. 10.1007/s00404-015-3982-9 26688284 PMC4908158

[53] Gonzalez-Covarrubias V., et al, Lipidomics of familial longevity. Aging Cell, 2013 12(3): p. 426–34. 10.1111/acel.12064 23451766 PMC3709127

[54] Mielke M.M., et al, The Association Between Plasma Ceramides and Sphingomyelins and Risk of Alzheimer's Disease Differs by Sex and APOE in the Baltimore Longitudinal Study of Aging. Journal of Alzheimer's disease: JAD, 2017 60(3): p. 819–828. 10.3233/JAD-160925 28035934 PMC5493514

[55] Nebel R.A., et al, Understanding the impact of sex and gender in Alzheimer's disease: A call to action. Alzheimers Dement, 2018. 10.1016/j.jalz.2018.04.008 PMC6400070 29907423

[56] Jack C.R. Jr., et al, Age, Sex, and APOE epsilon4 Effects on Memory, Brain Structure, and beta-Amyloid Across the Adult Life Span. JAMA Neurol, 2015 72(5): p. 511–9. 10.1001/jamaneurol.2014.4821 PMC4428984 25775353

[57] Mielke M.M., Vemuri P., and Rocca W.A., Clinical epidemiology of Alzheimer’s disease: assessing sex and gender differences. Clinical Epidemiology, 2014 **6**: p. 37–48. 10.2147/CLEP.S37929 PMC3891487 24470773

[58] Manjareeka M., et al, Correlation between anthropometry and lipid profile in healthy subjects of Eastern India. Journal of Mid-Life Health, 2015 6(4): p. 164–168. 10.4103/0976-7800.172302 26903756 PMC4743278

[59] Pasco J.A., et al, Prevalence of obesity and the relationship between the body mass index and body fat: cross-sectional, population-based data. PLoS One, 2012 7(1): p. e29580 10.1371/journal.pone.0029580 22253741 PMC3258232

[60] Adams T.D., et al, The relationship between body mass index and per cent body fat in the severely obese. Diabetes Obes Metab, 2007 9(4): p. 498–505. 10.1111/j.1463-1326.2006.00631.x 17587392

[61] Barber M.N., et al, Plasma Lysophosphatidylcholine Levels Are Reduced in Obesity and Type 2 Diabetes. PLOS ONE, 2012 7(7): p. e41456 10.1371/journal.pone.0041456 22848500 PMC3405068

[62] Heimerl S., et al, Alterations of plasma lysophosphatidylcholine species in obesity and weight loss. PLoS One, 2014 9(10): p. e111348 10.1371/journal.pone.0111348 25340546 PMC4207804

[63] Heffernan A.L., et al, The Neurobiology and Age-Related Prevalence of the ε4 Allele of Apolipoprotein E in Alzheimer’s Disease Cohorts. Journal of molecular neuroscience: MN, 2016 60(3): p. 316–324. 10.1007/s12031-016-0804-x 27498201 PMC5531868

