# Supplementary material for: Plasma lipidome variation during the second half of the human lifespan is associated with age and sex but minimally with BMI
Source: PLoS One. 2019 Mar 20;14(3):e0214141. doi: 10.1371/journal.pone.0214141 (PMC6426235; doi:10.1371/journal.pone.0214141)
Supplement: S1 Appendix — (DOCX) [file pone.0214141.s001.docx]

**S1 Appendix. Supporting Methods.**

LC MS/MS quality control samples

A set of quality control “standards” were injected every 20 runs. These controls included: (i) blank, to check for column and chromatography background levels, (ii) internal standards only, to check on system performance across a long sequence of runs, (iii) quality control plasma, to check for between run performance and enable calculation of between run and within run assay CV%.

Lipidsearch v4.1 search parameters

We performed search on raw files using the databases “General” and “labelled standards”. For peak detection, recalc isotope was set to “ON”, RT interval = 0.0 min. We used product search for LC-MS method and the precursor and product tolerances were set at 5.0 ppm and 8.0 ppm respectively. The intensity threshold was 1% parent ion, and the m-score threshold was set to 2.0. For quantitation, mz tolerance was set at -5.0 ppm to 5.0 ppm, and the retention time range was set at -0.5 to 0.5 min. The m-score threshold was 5.0, and all lipid classes were selected for inclusion. Ion adducts included +H, +NH4 for positive ion mode and –H, +HCOO for negative ion mode.
