# Supplementary material for: Plasma lipidome variation during the second half of the human lifespan is associated with age and sex but minimally with BMI
Source: PLoS One. 2019 Mar 20;14(3):e0214141. doi: 10.1371/journal.pone.0214141 (PMC6426235; doi:10.1371/journal.pone.0214141)
Supplement: S1 Table — (DOCX) [file pone.0214141.s002.docx]

**S1 Table. Lipid classes and number of lipids analysed in positive and negative ion mode.**

| **Plasma Lipid Category** | **Lipids analysed** |
| --- | --- |
| *Positive Ion Mode* | |
| Cer(d18:0/X) | 5 |
| Cer(d18:1/X) | 11 |
| CE(18:X) | 6 |
| CE | 20 |
| CE(20:X) | 4 |
| DG(16:0/X) | 4 |
| DG(18:0/X) | 3 |
| DG(18:1/X) | 7 |
| DG | 45 |
| LPC | 17 |
| PC(16:0/X) | 3 |
| PC(18:0/X) | 3 |
| PC(34:X) | 17 |
| PC(38:X) | 18 |
| PE(16:0/X) | 13 |
| PE(18:0/X) | 28 |
| PE | 50 |
| PS | 5 |
| SM(d18:1/X) | 16 |
| SM | 28 |
| TG | 229 |
| *Negative ion mode* | |
| LPC | 9 |
| LPE | 6 |
| PE | 40 |
| PI | 22 |
| PS | 9 |
| Total (both modes) | 618 |

Total list of lipids was produced via search and alignment on Lipidsearch v4.1, after appropriate filtering (see Supporting and main Methods). Negative ion mode produced similar associations with age as that of positive ion mode for the same classes.

Abbreviations: ceramide (Cer), cholesterol ester (CE), diacylglycerol (DG), lysophosphatidylcholine (LPC), lysophosphatidylethanoloamine (LPE), phosphatidylcholine (PC), phosphatidylethanolamine (PE), phosphatidylinositol (PI), phosphatidylserine (PS), sphingomyelin (SM), triglyceride (TG).
