# Supplementary material for: Plasma lipidome variation during the second half of the human lifespan is associated with age and sex but minimally with BMI
Source: PLoS One. 2019 Mar 20;14(3):e0214141. doi: 10.1371/journal.pone.0214141 (PMC6426235; doi:10.1371/journal.pone.0214141)
Supplement: S2 Table — (DOCX) [file pone.0214141.s003.docx]

**S2 Table. Patient characteristics and lipid profiles by age cohort.**

|  | HCS (56-75 yrs) | MAS (>75-95 yrs) | SCS(>95-99 yrs) | Chi-square |
| --- | --- | --- | --- | --- |
| N | 40 | 40 | 20 | N/A |
| Age | 63.2 (5.6) | 83.1 (4.8) | 96.6 (1.4) | 85.61* |
| BMI | 28.3 (5.2) | 28.1 (5.1) | 26.6 (5.8) | 1.159 |
| Lipid-lowering medication | 7 (17.5%) | 14 (35%) | 5 (25%) | 5.840 |
| Years of Education | 11.9 (1.2) | 10.9 (4.0) | 10.1 (3.5) | 7.244* |
| MMSE score | 28.3 (1.2) | 29.2 (0.76) | 26.04 (3.5) | 20.85* |
| WHR | 0.88 (0.10) | 0.93 (0.09) | N/A | 3.696 |
| LDL-C (mmol/L) | 3.22 (0.94) | 3.03 (1.05) | 2.94 (1.06) | 1.397 |
| HDL-C (mmol/L) | 1.38 (0.36) | 1.39 (0.34) | 1.47 (0.38) | 0.333 |
| Total Cholesterol (mmol/L) | 5.27 (0.85) | 4.99 (1.18) | 4.93 (1.10) | 2.080 |
| Triglycerides (mmol/L) | 1.31 (0.89) | 1.22 (0.63) | 1.13 (0.42) | 0.025 |

Abbreviations: body mass index (BMI), mini-mental exam (MMSE), waist-hip ratio (WHR), low density lipoprotein cholesterol (LDL-C), high density lipoprotein cholesterol (HDL-C).

Values represent mean (SD). * p<0.05; Kruskall Wallis test was used for all variables except the use of Lipid-lowering medications, in which case the Chi-square test for equality of proportions was used.
