# Supplementary material for: Plasma lipidome variation during the second half of the human lifespan is associated with age and sex but minimally with BMI
Source: PLoS One. 2019 Mar 20;14(3):e0214141. doi: 10.1371/journal.pone.0214141 (PMC6426235; doi:10.1371/journal.pone.0214141)
Supplement: S3 Table — (DOCX) [file pone.0214141.s004.docx]

**S3 Table. Patient characteristics and lipid profiles by sex.**

|  | **Males** | **Females** | **U** |
| --- | --- | --- | --- |
| N | 46 | 54 | N/A |
| Age | 78.6 (13.8) | 77.2 (13.8) | 1163.0 |
| BMI | 28.0 (5.3) | 27.7 (5.3) | 1185.5 |
| Lipid-lowering medication | 14 (30.4%) | 12 (22.5%) | N/A |
| Years of Education | 11.7 (3.8) | 10.7 (2.5) | 1061.5 |
| MMSE score | 28.3 (1.7) | 28.1 (2.4) | 1164.0 |
| WHR | .95 (.06) | .86 (.10) | 224.5* |
| LDL-C (mmol/L) | 2.82 (1.05) | 3.34 (.91) | 713.0* |
| HDL-C (mmol/L) | 1.31 (0.35) | 1.46 (.34) | 778.5* |
| Total Cholesterol (mmol/L) | 4.84 (1.09) | 5.33 (.94) | 797.0* |
| Triglycerides (mmol/L) | 1.38 (.88) | 1.13 (.56) | 865.5 |

Abbreviations: body mass index (BMI), mini-mental state examination (MMSE), waist-hip ratio (WHR), low density lipoprotein cholesterol (LDL-C), high density lipoprotein cholesterol (HDL-C).

Values represent mean (SD). *p<0.05 between sexes, Mann-Whitney U test.
