# Supplementary material for: Plasma lipidome variation during the second half of the human lifespan is associated with age and sex but minimally with BMI
Source: PLoS One. 2019 Mar 20;14(3):e0214141. doi: 10.1371/journal.pone.0214141 (PMC6426235; doi:10.1371/journal.pone.0214141)
Supplement: S4 Table — Correlations were taken for all subjects, then after correcting for sex, BMI and lipid-lowering medication usage, and after excluding those on lipid-lowering medication. (DOCX) [file pone.0214141.s005.docx]

**S4 Table: Correlations of lipid class normalised abundances with age.**

| **Plasma Lipid Category** | **r (all subjects)** | **r (all subjects, corrected)** | **r (all subjects, without meds)** |
| --- | --- | --- | --- |
| Cer(d 18:0/X) | -.500^a^ | -.552^a^ | -.416^b^ |
| Cer(d 18:1/X) | -.480^a^ | -.432^a^ | -.414^b^ |
| CE(18:X) | -.610^a^ | -.525^a^ | -.542^b^ |
| CE(20:X) | -.630^a^ | -.573^a^ | -.553^b^ |
| CE | -.560^a^ | -.546^a^ | -.555^b^ |
| DG(16:0/X) | -.140^b^ | -.185^b^ | -.155 |
| DG(18:0/X) | .010 | .041 | .013 |
| DG(18:1/X) | -.190 | -.153 | -.162 |
| DG | -.200 | -.161 | -.158 |
| LPC | -.580^a^ | -.589^a^ | -.549^b^ |
| PC(16:0/X) | -.260 | -.175 | -.115 |
| PC(18:0/X) | -.400^a^ | -.329^a^ | -.325^b^ |
| PC(34:X) | -.500^a^ | -.401^a^ | -.340^b^ |
| PC(38:X) | -.510^a^ | -.485^a^ | -.427^b^ |
| PE(16:0/X) | -.590^a^ | -.489^a^ | -.365^b^ |
| PE(18:0/X) | -.610^a^ | -.479^a^ | -.416^b^ |
| PE | -.450^a^ | -.477^a^ | -.442^b^ |
| PS | -.250 | -.130 | -.104 |
| SM(d 18:1/X) | -.570^a^ | -.578^a^ | -.532^b^ |
| SM | -.560^a^ | -.574^a^ | -.567^b^ |
| TG | -.420^a^ | -.320^a^ | -.372^b^ |

r is the Pearson product moment correlation coefficient of normalised lipid abundance with age. Correlations were taken for all subjects, then after correcting for sex, BMI and lipid-lowering medication usage, and then for all subjects, but excluding subjects on lipid lowering medications.

^a^ p<0.01, ^b^ p<0.05.
