# Supplementary material for: Plasma lipidome variation during the second half of the human lifespan is associated with age and sex but minimally with BMI
Source: PLoS One. 2019 Mar 20;14(3):e0214141. doi: 10.1371/journal.pone.0214141 (PMC6426235; doi:10.1371/journal.pone.0214141)
Supplement: S1 Fig — (DOCX) [file pone.0214141.s006.docx]

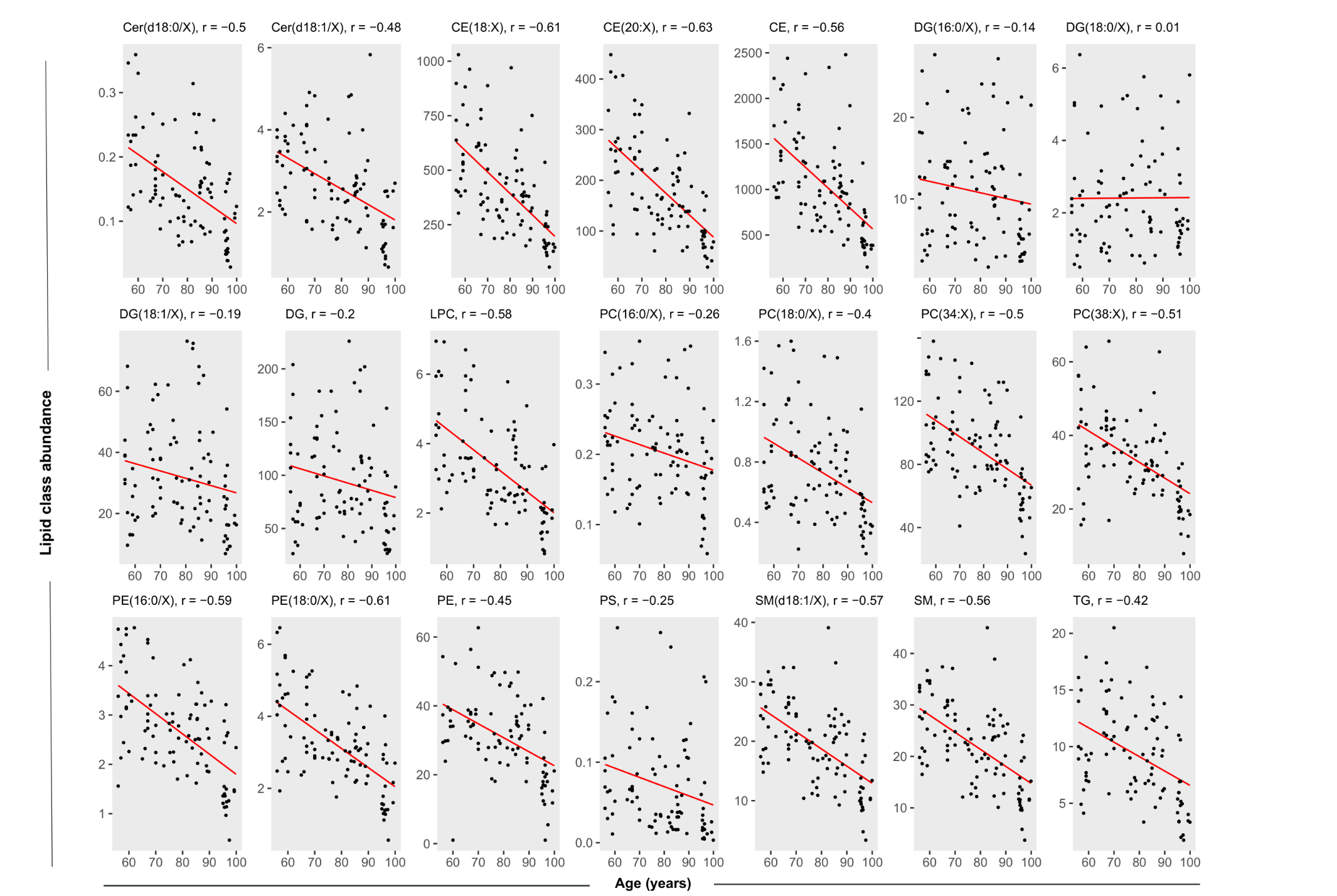


**S1 Fig. Scatterplot of age (years) with normalised lipid abundances for each lipid category.** Correlations are derived from the Pearson’s product moment coefficient. All r < -0.20 are considered significant at p<0.05
